# Clinical characteristics and follow-up results of 12 cases of paradoxical embolism

**DOI:** 10.1016/j.dib.2017.03.007

**Published:** 2017-03-11

**Authors:** Hong-liang Zhang, Zhi-hong Liu, Qin Luo, Yong Wang, Zhi-hui Zhao, Chang-ming Xiong

**Affiliations:** Center for Pulmonary Vascular Diseases, State Key Laboratory of Cardiovascular Disease, Fuwai Hospital, National Center for Cardiovascular Diseases, Chinese Academy of Medical Sciences and Peking Union Medical College, Beijing 100037, People׳s Republic of China

## Abstract

This article contains the clinical characteristics of the paradoxical embolized patients and the following up results. Data included are related to the article “Paradoxical embolism: Experiences from a single center” (Zhang Hong-liang, Liu Zhi-hong, Luo Qin, Wang Yong, Zhao Zhi-hui, Xiong Chang-ming, in press) [1]. The data are obtained from the hospital records and telephone interview.

**Specifications Table**

**Table:** 

Subject area	*Biology*
More specific subject area	*Medicine*
Type of data	*Table*
How data was acquired	*survey*
Data format	*Raw*
Experimental factors	*Medical records were retrieved for* paradoxical embolism.
Experimental features	*Clinical characteristics of the patients were collected from the medical records and follow-up data were recorded from hospital records or telephone interview.*
Data source location	*Center for Pulmonary Vascular Disease, Fuwai Hospital, Beijing, China*
Data accessibility	*Data is presented with this article.*

**Value of the data**

• These data provide the clinical characteristics and follow-up results of paradoxical embolism.
• Most patients had one or more risk factors for thrombosis and inducing or exacerbating factor.
• Long term coagulation is necessary and effective for paradoxical embolism.

## 1 Data

The data presented include the clinical characteristics of the paradoxical embolized patients, the administrated treatment and the following up results (Table 1).

## 2 Experimental design, materials and methods

Twelve patients diagnosed of paradoxical embolism in Fuwai Hospital from January 1994 to October 2015 were included. All clinical data related were collected from medical records, such as (1) demographic data; (2) case history and risk factors for thromboembolism; (3) clinical, laboratory and imaging findings of deep venous thrombosis, pulmonary embolism and systemic arterial embolic events; (4) imaging findings of an abnormal communication that allowed right to left shunt; (5) treatment and outcome; (6) follow-up data. Follow-up data were obtained from hospital records or by telephone interview with the patients or their family members. Follow-up ended on November 1st, 2015 [1].

## Conflict of interest

There was no conflict of interest to declare.

## Figures and Tables

**Table 1:** Clinical data and follow-up results of patients with paradoxical embolism.

**Case**	**Sex**	**Age, years**	**BMI, kg/m^2^**	**Risk factors for thrombosis**	**Inducing or exacerbating factors**	**Symptoms**	**DVT**	**PE**	**Systemic arterial embolism**	**Treatment**	**Outcome**	**Follow-up (years)**
1	M	59	–	CAG through femoral A, HT, smoking	Defecation	Syncope	R	Y	R CVA; mesentery A	CPR	Died	–
2	M	56	23.6	RF ablation through femoral A and V, smoking	Defecation	Syncope	B	Y	R renal A	CPR, warfarin	NA	NA
3	M	34	23.7	Smoking	–	Dyspnea, haemoptysis	L	Y	Thrombus straddled PFO	Thrombectomy, IVCF, warfarin	Alive	17.7
4	M	55	22.9	History of L lower limb bruising, HT	Sitting stilly for 2 h	Dyspnea, abdominal and lower back pain	L	Y	R renal A, mesentery A	Urokinase, warfarin, IVCF	NA	NA
5	F	75	23.8	Venous varices in B lower limbs, HT, smoking	Defecation	Dyspnea, palpitation, CVA	L	Y	L CVA	Urokinase, warfarin	NA	NA
6	M	51	18.9	Long-time standing work, smoking	Defecation	Dyspnea, convulsion	–	Y	B CVA	Urokinase, warfarin	Alive	13.7
7	F	27	21.0	Peripartum	Delivery	Dyspnea, palpitation, pain in B lower limb, CVA	B	Y	R CVA	Urokinase, warfarin, IVCF	Alive	13.5
8	M	39	24.8	–	–	Dyspnea, pain in L lower limb	R	Y	L popliteal A	rt-PA, warfarin	Alive	12.6
9	M	58	22.6	Sedentary lifestyle, hypercholesterolemia	Driving for 4 h	Dyspnea	L	Y	Descending aorta thrombus	rt-PA, warfarin	Alive	11.9
10	F	41	26.7	Overweight	–	Dyspnea, CVA	–	Y	L CVA	Warfarin	Alive	10.9
11	F	58	21.8	Venous varices in L lower limb	Defecation	Dyspnea, cough, CVA	–	Y	L CVA	Warfarin	Alive	10.8
12	F	42	27.1	Overweight	–	Dyspnea, CVA	–	Y	R CVA	Warfarin, pulmonary endarterectomy	Alive	10.6

**Abbreviations and illustration:** A, artery; B, bilateral; BMI, body mass index; CAG, coronary angiogram; CPR, cardiopulmonary resuscitation; CVA, cerebrovascular accident; DVT, deep venous thrombosis; F, female; HT, hypertension; IVCF, inferior vena cava filter; L, left; M, male; NA, nonavailable; PE, pulmonary embolism; R, right; RF, radiofrequency; Y, yes.

BMI of case 1 was unavailable.
